# Wool Keratin Hydrolysates for Bioactive Additives Preparation

**DOI:** 10.3390/ma14164696

**Published:** 2021-08-20

**Authors:** Carmen Gaidau, Maria Stanca, Mihaela-Doina Niculescu, Cosmin-Andrei Alexe, Marius Becheritu, Roxana Horoias, Cristian Cioineag, Maria Râpă, Ioana Rodica Stanculescu

**Affiliations:** 1 Leather Research Department, Research and Development National Institute for Textiles and Leather-Division Leather and Footwear Research Institute, 93, Ion Minulescu Street, 031215 Bucharest, Romania; carmen.gaidau@icpi.ro (C.G.); mihaela.niculescu@icpi.ro (M.-D.N.); cosmin.alexe@icpi.ro (C.-A.A.); 2 Doctoral School of Faculty of Applied Chemistry and Materials Science, Department of Science and Engineering of Oxide Materials and Nanomaterials, Politehnica University of Bucharest, 1-7 Polizu Street, 011061 Bucharest, Romania; 3 Probstdorfer Saatzucht Romania SRL, 20 Siriului Street, District 1, 014354 Bucharest, Romania; marius.becheritu@probstdorfer.ro (M.B.); roxana.horoias@probstdorfer.ro (R.H.); cristian.cioneag@probstdorfer.ro (C.C.); 4 Centre for Research and Eco—Metallurgical Expertise, POLITEHNICA University of Bucharest, 313 Splaiul Independentei, 060042 Bucharest, Romania; rapa_m2002@yahoo.com; 5 Horia Hulubei National Institute of Research and Development for Physics and Nuclear Engineering, 30 Reactorului Str., 077125 Magurele, Romania; istanculescu@nipne.ro; 6 Department of Physical Chemistry, University of Bucharest, 4–12 Regina Elisabeta Bd., 030018 Bucharest, Romania

**Keywords:** keratin hydrolysates, enzymatic hydrolyses, bioactive keratin, organised protein structures

## Abstract

The aim of this paper was to select keratin hydrolysate with bioactive properties by using the enzymatic hydrolysis of wool. Different proteolytic enzymes such as Protamex, Esperase, and Valkerase were used to break keratin molecules in light of bioactive additive preparation. The enzymatic keratin hydrolysates were assessed in terms of the physico-chemical characteristics related to the content of dry substance, total nitrogen, keratin, ash, cysteic sulphur, and cysteine. The influence of enzymatic hydrolysis on molecular weight and amino acid composition was determined by gel permeation chromatography (GPC) and gas chromatography-mass spectrometry (GC-MS) analyses. Antimicrobial activity of keratin hydrolysates was analysed against *Fusarium* spp., a pathogenic fungus that can decrease the quality of plants. The bioactivity of enzymatic hydrolysates was tested on maize plants and allowed us to select the keratin hydrolysates processed with the Esperase and Valkerase enzymes. The ratio of organised structures of hydrolysate peptides was analysed by attenuated total reflectance-Fourier transform infrared (ATR-FTIR) deconvolution of the amide I band and may explain the difference in their bioactive behaviour. The most important modifications in the ATR spectra of maize leaves in correlation with the experimentally proven performance on maize development by plant length and chlorophyll index quantification were detailed. The potential of enzymatic hydrolysis to design additives with different bioactivity was shown in the case of plant growth stimulation.

## 1. Introduction

Biomass containing keratin is generated by the food industry, slaughterhouses, wool industry, and textiles at the end of their life cycle, with keratin being considered as one of the most abundant biopolymers [1]. Even the potential of keratin-based biomass use is significant; at present, it is burned, with a high energy consumption and harmful gas release [2]. The natural decomposition of feather and wool generates a bad odour, methane, and carbon dioxide, which are classified as greenhouse gases [3]. At the EU level, a resource of 150,000 tons of unmarketable wool, which represents about 75% of total wool production per year, becomes waste.

Wool is an excellent animal fibre composed of 82% hard keratin (α-keratin) with outstanding chemical and enzyme attack resistance due to the hierarchical structure of polypeptides stabilised in helical shape by hydrogen and hydrophobic bonds. Intramolecular and intermolecular cystine bonds are also responsible for keratin insolubility and slow natural biodegradability.

The main methods for keratin extraction and solubilisation are classified [4] as chemical (reduction, oxidation, hydrolysis, sulphitolysis, assisted by ionic liquid), physical (steam explosion, superheated water treatment, supercritical water extraction, and microwave irradiation) and biological (microbial and enzy matic).

The enzymatic and bioconversion methods that approach milder reaction conditions are considered to preserve functional properties of keratin [5]. The extracted keratin protein is composed of three main fractions originated from the wool fibre cortex, with high molecular weight and low-sulphur protein, and from the interfilament matrix with middle molecular weight and high-sulphur content, and with low molecular weight, rich in glycine/tyrosine amino acids [6].

The use of enzymes for keratin hydrolysis allows selecting and tailoring suitable functionalities for biotechnological applications [5]. The keratin potential for cell adhesion and keratinocyte proliferation has been demonstrated in wound dressing fabrication and medical testing [7,8]. The keratin extracted using keratinase showed 1.5-fold cell viability in biocompatibility tests with L929 mouse fibroblasts and wound healing efficiency, superior to chitosan. The bioactivity of keratin was attributed to the peptide-binding motifs namely LDV (Leu-Asp-Val), EDS (Glu-Asp-Ser), and RGD (Arg-Gly-Asp) from which growth factors bind to fibroblast and trigger cell proliferation and migration [9].

Other research has shown the positive influence of feather keratin hydrolysates on rhizobacteria soil enrichment, different plant biofertilisation, and the favourable impact on human health and the ecosystem [10]. The composition of keratin hydrolysates in free amino acids and oligopeptides [11] with the role of organic nitrogen source, growth inducers through hormone-like action, may explain the fertilisation and biostimulation efficiency in foliar applications [12]. The use of wool keratin hydrolysates in grasslands fertilisation can close the loop cycle of carbon and increase the carbon sequestration rate. The role of keratin hydrolysate in soil degradation amending, water conservation, and pesticide retention was underlined as a sustainable solution that can be used on site [13]. The seed treatment of *Lepidium Sativum* plants with the keratin hydrolysate extracted in a pilot superheat water equipment showed an increased number of sprouted seeds by 11.9% and higher root length by 58.2% [14]. The use of bovine hair keratin hydrolysate in coating compositions for urea pelleting as a slow-release soil fertiliser preparation was recently reported [15]. *Trichoderma asperellum* was grown in wool and feather wastes and the mixture of fungi and keratin metabolites showed potential for increased plant health and productivity in tomatoes. According to reported research [16], the wool keratin hydrolysate was shown to be more of a biostimulant by increasing the tomatoes’ plant biomass compared to the feather keratin hydrolysate with a biostimulant effect only on plant size. There have been few reports on wool keratin as a foliar fertiliser [4] and the correlation with keratin hydrolysate properties.

In this paper, the enzymatic hydrolysis of wool alkaline keratin hydrolysate with three proteases, and the specific influence on chemical, structural, and bioactive properties are shown with the prospect of finding applications for more sustainable agriculture. The research hypothesised that the keratin hydrolysates with lower molecular weights with still secondary structured peptides, free amino acid, sulphonic, and sulphonated cysteine composition are an efficient bioactive product compared to higher molecular weight keratin hydrolysates. The bioactive functionality of alkaline-enzymatic keratin hydrolysate was successfully experimented on foliar fertilisation of maize plants with significant influence on plant growth compared to the untreated plants.

## 2. Materials and Methods

### 2.1. Materials

Wool was purchased from sheep farmers from Romania. Chemical reagents of analytical grade like sodium hydroxide, ammonia (25%), sodium carbonate, formic acid, and sulphuric acid were purchased from Chemopar SA (Bucharest, Romania). Borron SE (ethoxylated alkyl derivatives with 65% concentration) was supplied by SC Triderma SRL (Bucharest, Romania). Promatex^®^, an endo-protease from *Bacillus spp* with the activity of 1.5 AU-A/g, in the range of pH = 6–9 and temperature of 30–65 °C and Esperase^®^ 8.0 L, a serine endo-peptidase from *Bacillus lentus* with the activity of 8 KNPU-E/g, working at elevated temperature and pH = 8–12.5, were purchased from Novozymes (Atasehir, Turkey). Valkerase^®^, a keratinase, serine protease from *B. licheniformis,* with activity of 80,549 U/g at pH = 5.5 and 55 °C, was supplied by BioResource International (Durham, NC, USA).

Maize seeds (*Zea mays* L.) were supplied by Probstdorfer Saatzucht Romania SRL (Bucharest, Romania), namely the Olt hybrid, FAO 430, the same one used in the field, in the micro- and macroplots.

### 2.2. Keratin Hydrolysates Preparation

Alkaline hydrolysate was prepared by a previously described method [17]. To obtain the alkaline hydrolysate, 1.2 kg wool was pretreated by washing, degreasing using 4% w/w NH_4_OH, 0.6% w/w Borron SE, and 1% w/w Na_2_CO_3_ for 2 h at 40 °C, washing up to neutral pH, cutting with a bench grinding machine (La Minerva, Minerva Omega, Bologna, Italy), and then mixed with 6 L of 2.5% w/v sodium hydroxide solution in a stainless steel vessel equipped with a mechanical stirrer and automatic temperature control (SC Caloris SA, Bucharest, Romania) at 80 °C, for 4 h, when wool was solubilised with 96.5% yield. The remaining 3.5% insoluble cell membrane complex originated from the wool fibre cuticle and cortex [18] is also a biodegradable product. To obtain keratin hydrolysates with different characteristics, the alkaline hydrolysate was enzymatically hydrolysed using 1% w/w concentration of three enzymes: Protamex, Esperase, and Valkerase under optimum activity conditions for 4 h. All keratin hydrolysates were conditioned at pH = 7 with sulphuric acid. The enzymatic keratin hydrolysates were labelled PRO, ESPE, VALKE, and the alkaline keratin hydrolysate HK. Lyophilised variants were performed by freeze drying of keratin dispersions in a DELTA 2-24 LSC freeze-dryer, laboratory scale (Osterode am Harz, Germany).

### 2.3. Keratin Hydrolysates Characterisation

Physico-chemical characterisations were performed according to the standard in force or in house methods: dry substances (SR EN ISO 4684:2006), ash (SR EN ISO 4047:2002), total nitrogen and protein content (SR EN ISO 5397:1996), aminic nitrogen (ICPI method), cysteine (SR 13208:1994), cystine sulphur (SR 13208:1994) content, and pH (STAS 8619/3:1990).

The molecular weights were determined by gel permeation chromatography (GPC) using an Agilent Technologies instrument (1260 model) (Agilent Technologies, Santa Clara, CA, USA) equipped with PL aqua gel-OH MIXED-H column (7.5 × 300 mm, 8 µm) and multi-detection unit. Optimum working conditions for GPC were flow rate of mobile phase containing 1 mL min^−^^1^, injection volume of the sample 100 µL, and temperature of 35 °C for the detectors and column. Calculations of the Mw and number average molecular weight (Mn) were performed with the Agilent GPC/SEC Software (Version 1.1, Agilent Technologies, Santa Clara, CA, USA).

The amino acid analysis was performed using a GC-MS (Thermo Scientific TRACE 1310/TSQ 8000Evo, Waltham, MA, USA.) Samples were derivatised with acetonitrile and BSTFA at 105 °C. The GC separation was achieved using a capillary column TG-5SILMS (5% diphenyl/95% dimethyl polysiloxane), 30 m × 0.25 mm × 0.25 µm. The operating conditions for GC were an initial temperature of 100 °C raised to 170 °C (°C/min) then to 190 (3 °C/min) and the final temperature was 280 °C. The total run time was 40 min. The temperature for the transfer line was set at 280 °C and the ionisation source at 230 °C. Injection volume was 0.5 µL. The MS detector was operated in continuous scan mode with the m/z interval ranging from 40 to 600 amu. The data were analysed using the Chromeleon v.7.2.7 software. For quantification of amino acids, a calibration curve was performed.

The average particle size and zeta potential characteristics were measured using a Zetasizer Nano-ZS device (Malvern Instruments, Malvern, UK) on keratin dispersions without dilutions, at 25 °C, in triplicate.

FTIR measurements were performed using an INTERSPEC 200-X spectrophotometer (Interspectrum, Tartumaa, Estonia) equipped with an ATR device. The spectra of the samples were achieved in triplicate by examining the frequency range 4000–700 cm^−1^ with a 2 cm^−1^ resolution and placing the lyophilised keratin hydrolysates on a ZnSe crystal. Amide I band’s deconvolution was performed in order to identify the ordered structures affected by enzymatic hydrolyses and to correlate with the bioactive properties. Thus, β-sheets, random coils, and α-helix were examined in the ranges of 1613–1637 cm^−^^1^, 1637–1645 cm^−^^1^, and 1645–1662 cm^−^^1^, respectively [19]. The deconvolution parameters were settled in the spectra range of 1700–1600 cm^−^^1^ using a Lorrentian function. Secondary structure of wool keratin hydrolysates were obtained by using the ratio between the individual area band to the whole area bands.

### 2.4. Antimicrobial Activity Evaluation

Antimicrobial activity was determined according to an adapted absorption quantitative method involving the direct inoculation of *Fusarium* *oxysporum* ATCC 48112 on the keratin samples.

The stock culture of *Fusarium spp* was prepared by growing it in Sabouraud dextrose agar at 25 °C for up to 72 h. The initial cell concentration was 6.8 × 10^3^ CFU/mL. The mixture of inoculums with keratin hydrolysates was made in sterilised Eppendorf tubes and incubated for 24 h and then evenly poured on Petri dishes. After another 24 h of incubation at 25 °C, the CFUs were counted using a Funke Gerber counter (Funke Gerber, Surrey, UK). The antimicrobial efficiency was calculated and expressed as percent and logarithm of the CFUs’ reduction as an average of three parallel samples.

### 2.5. Bioactive Properties Test on Maize Seeds and Plants

The maize grain germination was prepared on five repetitions of 20 seeds immersed in water for the control and in enzymatic keratin dispersions. The maize plant seedlings were transferred in pots and were foliar fertilised at stages of 2–3 leaves, 5–6 leaves, and 6–8 leaves with water for the control and with water diluted enzymatic hydrolysates (1:6) for the tested samples. The plant length was recorded for up to 50 days as well as the foliar chlorophyll, which was assessed using a chlorophyll content meter type CCM-200 PLUS (Opti-Sciences Inc. Hudson, NY, USA). The second tests were performed at the experimental field level with selected products (ESPE, VALKE, water) in the second year on the maize seeds and plants, following the same protocol. ATR-FTIR spectroscopy investigation was performed directly on the leaf blade verso with a Bruker VERTEX 70 spectrometer equipped with diamond crystal ATR accessory, working in the 4000–600 cm^−1^ range with 64 scans and 4 cm^−1^ resolution in triplicate to investigate changes in the maize leaves’ chemical compositions. A KBr beam splitter and a RTDLaTGS (Room Temperature Deuterated Lanthanum α Alanine doped TriGlycine Sulphate) detector were used. Spectra were processed with the OPUS software using atmospheric compensation, vector normalisation, baseline correction with straight lines, and one iteration of additional concave rubber band correction and automatic peak peaking.

### 2.6. Statistical Analysis

The statistical processing was done using analysis of variance (ANOVA) (95% significant level) on each pair of interest and differences at *p* < 0.05 were considered statistically significant.

## 3. Results

### 3.1. Keratin Hydrolysate Preparation

**Figure 1:**
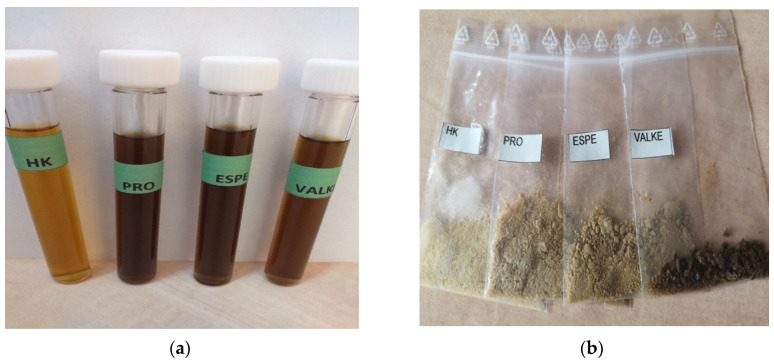
(**a**) Alkaline (HK) and enzymatic keratin hydrolysates (PRO, ESPE, VALKE); (**b**) lyophilised keratin hydrolysates (HK, PRO, ESPE, VALKE).

The alkaline keratin hydrolysate and enzymatic keratin hydrolysates in dispersion and in the lyophilised state are presented in Figure 1. The enzymatic hydrolysates were a darker colour compared to the alkaline keratin hydrolysate (Figure 1a) in dispersions and in lyophilized powders (Figure 1b).

### 3.2. Keratin Hydrolysates Characterisation

Physico-chemical analyses of keratin hydrolysates are presented in Table 1. It can be seen that the concentrations of keratin dispersions were similar for all versions as well as the ash content; the protein content was higher for ESPE and VALKE compared to HK. The aminic nitrogen values suggest that keratin molecule breaking occurs substantially more intensely in the VALKE sample, and the cysteine concentration and cystinic sulphur were higher in enzymatic hydrolysates compared to alkaline hydrolysate. The increases in cysteine concentration in enzymatic hydrolysates were probably due to the associative properties of peptides [11] and renowned heterogeneity of keratin, composed of three distinct fractions. Possible reversible oxidative reactions can occur in enzymatic hydrolyses, so cysteine and cystinic sulphur can increase in concentration [12]. Decreased concentrations in cysteine and cystinic sulphur were found in ESPE and VALKE compared to PRO in correlation with decreased molecular weights. The concentrations in free amino acids in the most interesting enzymatic hydrolysates, ESPE and VALKE, were found to be almost similar.

The free amino acid composition of enzymatic keratin hydrolysates was performed for the ESPE sample and showed that it was composed of 42.76% L-oxoproline-2TMS, 16.19% glutamic acid-3TMS, 11.93% glycine-3TMS, 10.55% L-alanine, 6.72% L-leucine, 5.14% glycine-2TMS, 2.29% L-valine, 1.59% L-proline, 1.57% L-isoleucine, and 1.23% L-treonine-2TMS. It is already known that some amino acids have a specific role in plant physiology: in amino acid synthesis (glutamic acid), stress condition resistance (leucine, L-proline) and respiratory metabolism (cysteine) etc.

**Figure 2:**
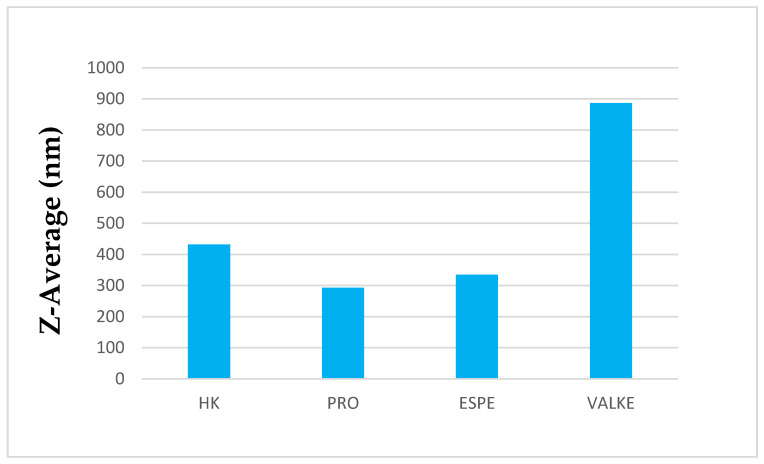
Average particle sizes of keratin hydrolysates.

**Figure 3:**
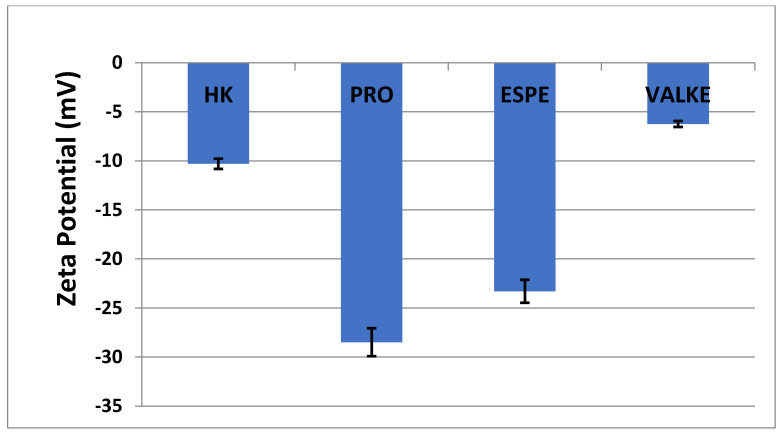
Zeta potential of keratin hydrolysates.

The average particle sizes of keratin dispersions are presented in Figure 2 and show that the enzymatic hydrolyses for the PRO sample led to lower average particle sizes compared to keratin alkaline hydrolysate HK, while the increased particle sizes were present in the most broken keratin samples (ESPE and VALKE). This behaviour may be explained as an effect of the properties of the self-assembling peptides: the sample VALKE, with the lowest molecular weight, showed the highest particle size, in correlation with the zeta potential with the lowest absolute value (Figure 3). The most stable dispersions were found to be the PRO and ESPE samples with close average particle sizes.

**Figure 4:**
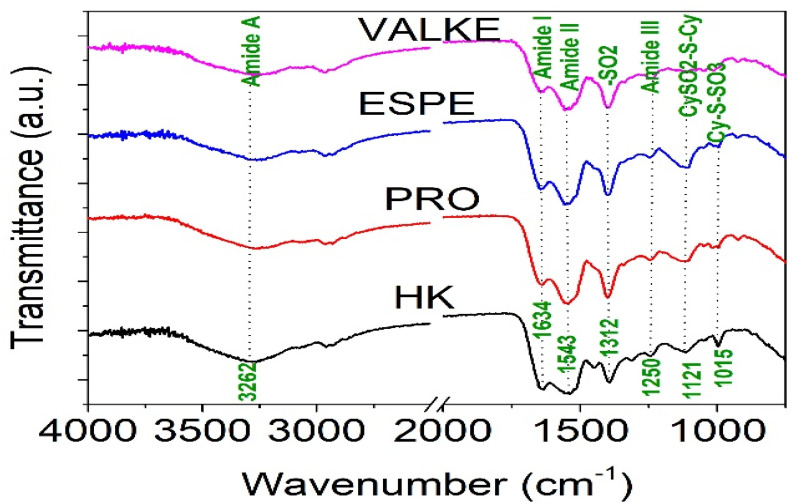
FTIR spectra for keratin hydrolysates: HK, PRO, ESPE, and VALKE.

**Figure 5:**
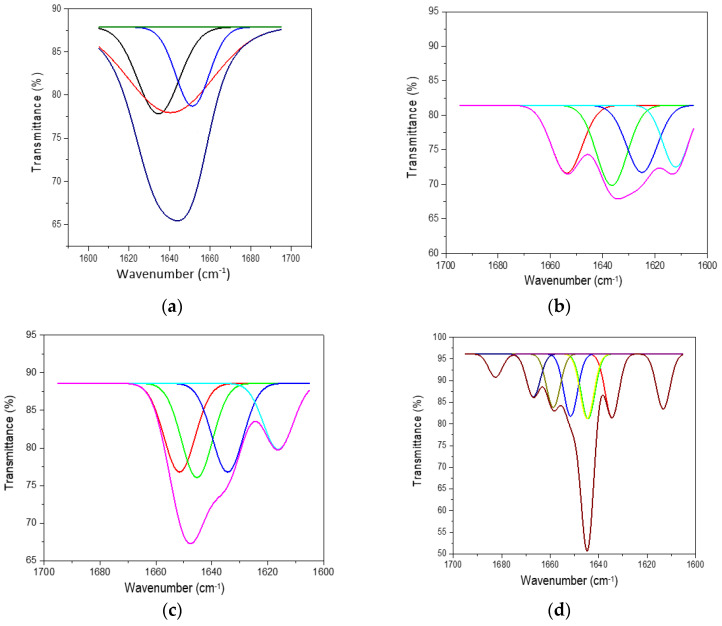
Deconvoluted amide I band for (**a**) HK, (**b**) PRO, (**c**) ESPE, and (**d**) VALKE.

The ATR-FTIR characterisation of lyophilised keratin hydrolysates processed by enzymatic hydrolyses was performed to identify the main functional groups (Figure 4) and organised structures ratio of keratin compared to the alkaline keratin hydrolysate by deconvolution of the amide I band (Figure 5, Table 2).

The amide A band connected to N–H stretching bonds was recorded for all keratin hydrolysates at 3262.5–3265.2 cm^−1^, which is an indication for polypeptide chain interacted by hydrogen bonds to form twisted β structural sheets.

The bands at 2958.6–2959.1 cm^−1^ and 2930–2932 cm^−1^ can be attributed to methyl antisymmetric and symmetric modes of proteins.

The amide I band correlated to the stretching vibration of the carbonyl groups was recorded at 1634.4 cm^−1^ (HK) and 1634.5 cm^−1^ (VALKE) for the β keratin sheets and at 1640.6 cm^−1^ (PRO), 1644.8 cm^−1^ (VALKE), and 1645 cm^−1^ (ESPE) for α keratin [19,20]. The amide II band was attributed to the N–H bending vibration and C–N stretching vibrations and was recorded at 1543.6–1558.6 cm^−1^. The bands in the region of 1300–1350 cm^−1^ were attributed to the sulphonamide type of grouping –SO_2_ and was found for HK (1312.3 cm^−1^) and shifted at 1342.6–1395.3 cm^−1^ for all analysed enzymatic keratin hydrolysates. Amide III band (1250 cm^−1^) was found for all analysed products at 1244.2–1248.3 cm^−1^, and CySO_2_–S–Cy (1121 cm^−1^) was found for keratin hydrolysates at 1121.9–1123.3 cm^−1^. The S–O symmetric stretch band attributed to Cy–S–SO_3_ (Bunte salt) at 1015.8–1021.1 cm^−1^ and S–O symmetric stretch attributed to cysteic acid at 1049.4–1051 cm^−1^ were found only in the enzymatic keratins, showing oxidative reactions compared to alkaline keratin. It is already known that Bunte salt is a plant metabolism precursor, which can explain the efficiency of enzymatic keratins in maize plant fertilisation. The vibration band at 995.6–997 cm^−1^, attributed to C–S–H was identified in all keratins with the exception of the VALKE product. Amide IV bands of the O=C−N vibration were found only in enzymatic keratins at 925.1–925.2 cm^−1^. It can be concluded that more oxidized groups can be found in VALKE keratin than in other keratin hydrolysates, in agreement with lower molecular weight (Table 1) and the darkest colour. It is known that endo-peptidase enzymes are able to break the protein molecules at the polypeptide chain level, and according to our research results, different levels of hydrolyses were performed with three kinds of proteases.

It was proven that the enzymatic cleaving of keratin sulphide bonds is similar to chemical sulphitolysis process due to the sulphites released by microorganisms according to the Reaction [21]:

*cys* − *SS* − *cys (cystine)* + *HSO*_3_** ⇔ *cySH (cysteine) + cyS.SO*_3_** *(S-sulphocysteine)* (1)

The air oxidation of more available sulphur amino acids from VALKE products led to the formation of cysteic acid [22]. The darker colour of the lyophilised keratins confirms the oxidative processes in enzymatic keratins with lower molecular weight (Figure 1).

Deconvolution of the amide I (1700–1600 cm^−1^) band of keratin hydrolysates allowed us to analyse the secondary structure of proteins and identify the potential influence on the bioactive properties. In Figure 5, the deconvoluted spectra of keratin hydrolysates are presented and confirm the specific features of different hydrolysates. In Table 2, the shares of β-sheets, α-helix, and random conformations are presented.

**Figure 6:**
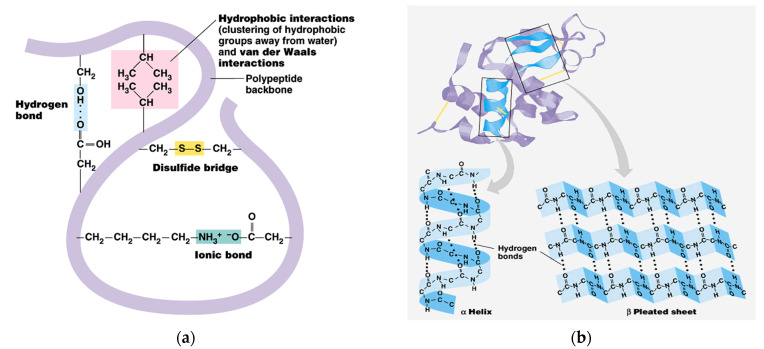
Keratin molecule with intra-molecular bonds (**a**) and transition of α-helix to β-sheets (**b**) [24].

The share of ordered structures was found to be HK ≥ PRO > ESPE > VALKE with decreased ratios, in agreement with the range of molecular weights (Table 1). Compared to the wool composition of 56% α-helix and 10% β-sheets and restructured wool with 47% α-helix and 33% β-sheets, keratin hydrolysates showed a decreased proportion of α-helix structures with decreased molecular weights and amorphous keratin components for ESPE and VALKE keratin hydrolysates. It is known that the transition of α-helix to β-sheets is made by molecule unfolding and broken hydrogen bonds. In the VALKE hydrolysate, many hydrophobic, disulphide, and ionic bonds were broken (Figure 6) and the helix was unfolded and restructured in β-sheets with more available contact surface. Compared to the wool and restructured wool with 66% and 80% [23], respectively, ordered structures shares, ESPE and VALKE showed 71.8% and 54.7%, respectively, which means that by enzymatic hydrolyses, the biocompatible protein structures are still available in a consistent share, with prospect for bioactive efficiency.

### 3.3. Antifungal Properties of Keratin Hydrolysates

It is well known that *Fusarium* spp. is a pathogenic fungus and the origin of important qualitative and quantitative yield loss in agriculture. Good agriculture management practice and fungicide use are the main tools in maize plant culture without important loss. The active ingredients of fungicides are known to induce pathogen resistance and their efficiency is connected to the suitable moment of application. The fungicides are in continuous revisions due to their involvement in endocrine disruption and toxicity.

The use of natural inducers of the plant’s self-defense system is already recognised as an ecological tool for maize plant protection [25]. The presence of sulphide, sulphoxide, and other derivates identified in keratin hydrolysates can explain the good resistance against *Fusarium* spp. (Table 3), all the more so as studies have indicated that the fungus has shown the highest affinity for wool compared to other strains [26].

### 3.4. Bioactive Keratin Hydrolysates for Plant Growth Biostimulation

**Figure 7:**
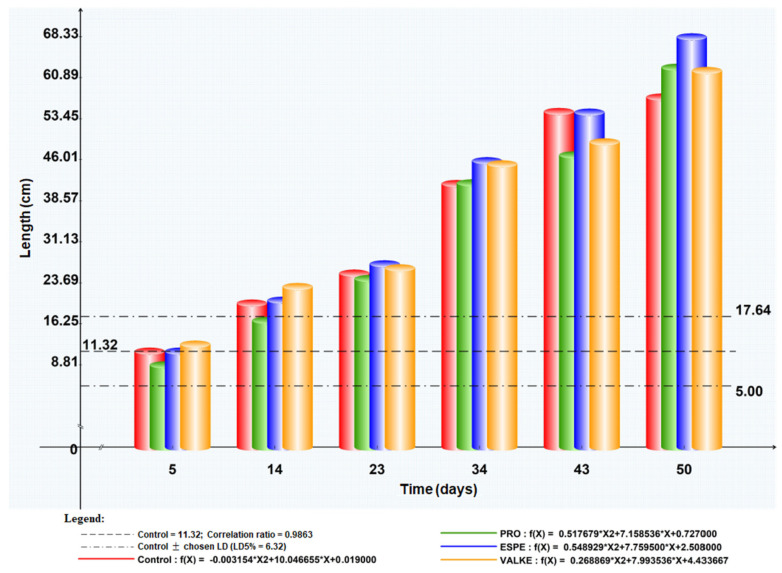
Maize plant length evolution after foliar fertilisation with keratin hydrolysates.

**Figure 8:**
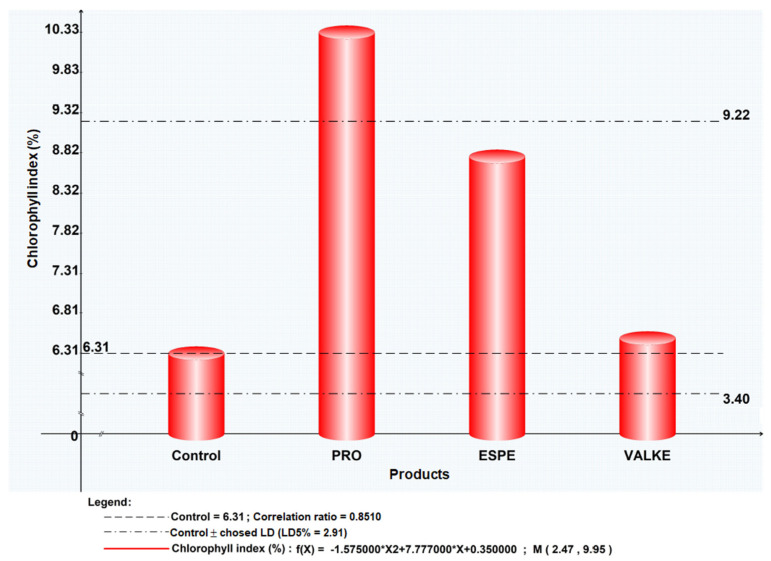
Chlorophyll index for maize plants treated with keratin hydrolysates.

The maize plant foliar fertilisation in laboratory and greenhouse conditions showed that the plant length was higher by 8.4–19% (Figure 7) as well as chlorophyll concentration, which increased compared to the control samples (Figure 8). Maize plant increase of 4% in length was recorded in reported experiments for fertilisation with protein hydrolysates from vegetable sources [27]. ESPE and VALKE products showed improved performances in plant growth stimulation, ESPE showing by far the highest performance with an influence on inflorescence and fructification compared to the other variants. The chlorophyll index was shown to be augmented for plants treated with enzymatic keratin hydrolysate compared to the control sample.

The general observations were that after foliar fertilisation with the enzymatic keratin hydrolysates, the plants showed higher adaptation to climate change and favoured inflorescence, especially in the case of the experiments with the ESPE and VALKE samples. The results suggest that lower molecular weights of keratin hydrolysate with available amino acids and still organized protein structures were more favourable for plant growth biostimulation.

**Figure 9:**
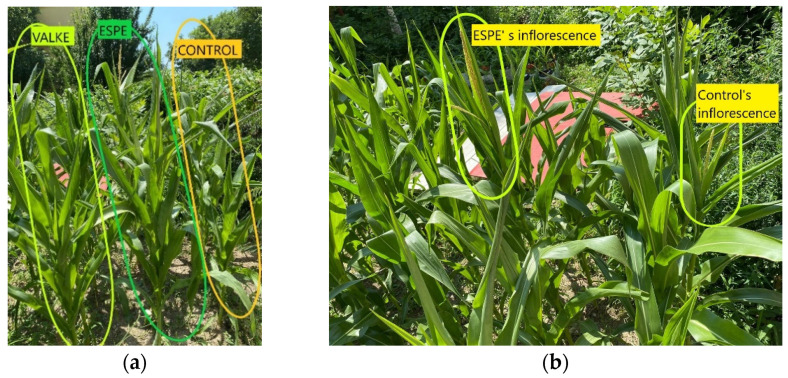
**(a**) The higher maize plants foliar fertilised with ESPE and VALKE products compared to the control maize plant; (**b**) the more developed inflorescence of maize plant treated with the ESPE product compared to the control plant.

The tests performed in the second year at the experimental field level by foliar fertilisation of maize plants with selected keratin hydrolysates, ESPE, and VALKE, following the same protocol of application set in the first year at the laboratory level, confirmed the biostimulation effect on plant growth compared to the control sample (Figure 9). The biostimulant effect of ESPE and VALKE products on maize plants could be observed compared to the control plant (Figure 9a) and had more developed inflorescence compared to the control sample (Figure 9b).

**Figure 10:**
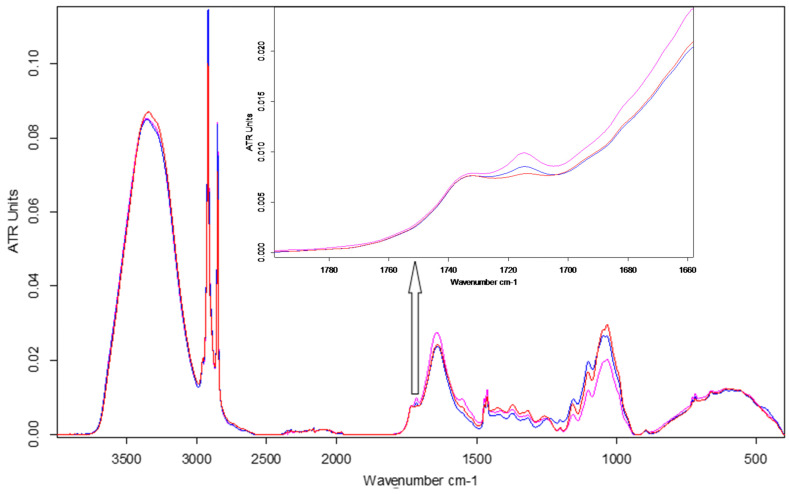
FTIR spectra of maize leaves: control, ESPE, and VALKE with details of the phenolic ester band at 1715 cm^−1^.

ATR-FTIR spectroscopy emphasised changes in the chemical fingerprint of ESPE and VALKE treated plants toward the control leaf samples (Figure 10), especially in the cellulose, protein, and phenolic composition as determined by modifications of band position, intensity, and shape. Average spectra from three determinations are reported.

The main assigned stretching (ν) and bending (δ) peaks identified in the ATR-FTIR spectra of leaves [28,29,30,31] are listed in the Table 4.

The leaves of plants treated with ESPE and VALKE hydrolysates showed evident structural differences by means of spectral modifications in terms of higher intensity bands compared to the control leaves: in the aliphatic region at 2917 and 2849 cm^−1^ and also in the protein region due to an enhancement of amide I at 1640 cm^−1^ and amide II at 1555 cm^−1^ bands. The obvious increase in the band at 1714 cm^−1^ suggested the appearance of an aromatic ester as a consequence of the treatment. The approx. 10 cm^−1^ blue shift of the NH stretching band could be due to conformational modification of protein structures in the treated leaves compared to the control. The ESPE hydrolysate showed the highest impact on maize development as can be seen from the highest shifts of band position and intensity toward the control, in comparison with the VALKE hydrolysate. Table 4 also shows an important shift in the position of the amide III band of about 16 cm^−1^ for the ESPE treated plants compared to the 1 cm^−1^ shift for the VALKE treated plants, indicating important modification of secondary protein structures. The most intense complex peak in the fingerprint region, attributed to cellulose structures, showed two maxima at about 1048 and 1034 cm^−1^ with the same intensity peaks for the ESPE plant series, while the VALKE and control series had different intensities as another consequence of the increased biostimulation of the ESPE keratin hydrolysate.

## 4. Discussion

Low grade wool can be efficiently solubilised, and bioactive components released through alkaline-enzymatic hydrolysis with significant efficiency in maize plant growth stimulation. The research demonstrated that a by-product from animal breeding activity can be integrated in agriculture, in the framework of the general concept of circular economy with a reduction in fossil fuel origin fertilisers and environmental pollution [32].

The alkaline hydrolysis of wool has been recognised as a simple and high yield method for the recovery of waste wool with the potential to preserve the basic structural characteristics of keratin [33] compared to other investigated methods. In our previous research [17], we showed that through alkaline hydrolysis, the waste wool can be completely solubilised and recovered. The present research showed that the alkaline keratin hydrolysates can be enzymatically processed with the purpose of tailoring low molecular weight keratins with the preservation of some still structured bioactive peptides.

The molecular weights of alkaline HK (38,099 Da), alkaline-enzymatic PRO (35,112 Da), and ESPE (12,400 Da) hydrolysates were higher compared to other reported alkaline hydrolysates of 3500–10,000 Da [34], showing a broader range of possibilities for keratin molecule tailoring for different purposes. The concentrations of free amino acids in ESPE and VALKE products were found to be 4.6% and 4.3%, respectively, reported to protein weight, which represents 3.5% and 3.1%, respectively, from the wool weight compared to other reported values of 18.8%, in the case of steam explosion solubilisation of keratin [35]. The compositions of free amino acids found in the ESPE product dominantly comprised specific amino acids for high- sulphur fractions (49.8%), with medium molecular weight, followed by amino acids specific for the low-sulphur fraction (31.2%) with high molecular weight and only 11.9% was represented by specific amino acids for the low molecular fraction [6]. The concentration in free amino acids of ESPE and VALKE is suitable for foliar fertilisers and applications in diluted state, which is an economical advantage compared with other formulations based on solid state proteins [28].

The average particle sizes of alkaline enzymatic keratin hydrolysates were lower compared to the alkaline keratin hydrolysate except for VALKE with the highest average particle size. The VALKE product had the lowest molecular weight, which suggests that the self-assembling properties led to the highest average particle size, in agreement with the lowest zeta potential, which shows that the particles present a limited water molecules layer, the potential being close to the isoelectric point with a value of −6 mV. The same tendency can be seen for ESPE with lower molecular weight compared to PRO and a slightly higher average particle size (293 nm compared to 334 nm) and lower zeta potential (−23.3 mV compared to –28.5 mV, respectively). The results showed that the performed hydrolyses were not as destructive for the keratin molecule as those reported for the enzymatic hydrolysates when the average particle size was reported to be 100–150 nm [36], but comparable to those of 750 nm, tested as a bioactive and biocompatible material for wound healing [34]. The self-assembling ability of keratin hydrolysate was also highlighted for keratin hydrolysates extracted by keratinase use [34] or by ultrasound assisted chemical extraction [37].

ATR-FTIR analyses allowed for the identification of the S–O symmetric stretch band attributed to Cy–S–SO_3_ (Bunte salt) at 1015.8–1021.1 cm^−1^ and the S–O symmetric stretch attributed to cysteic acid at 1049.4–1051 cm^−1^ in only alkaline-enzymatic keratins, which can explain the improved bioactivity of selected hydrolysates. The amide I deconvolution results revealed that the alkaline-enzymatic keratins had still secondary structured peptides of α-helix (26.53–27.11%) and β-sheets (26.53–45.30%) with a lower concentration in VALKE, in agreement with its lower molecular weight. The colour change in the alkaline-enzymatic keratin dispersions and lyophilised powders compared to alkaline keratin was attributed to the release of chromophores from the aromatic amino acids such as tyrosine and phenylalanine [34] and confirms the oxidative processes identified by spectral analyses. The presence of cystinic sulphur in all keratin hydrolysates explains the antifungal activity against *Fusarium oxysporum* ATCC 48112, with a protective role on plant growth, which agrees with other studies related to the outstanding antioxidant properties of keratin and reactive oxygen species generation in a comparable measure such as vitamin C [9].

The maize plant foliar fertilisation with keratin hydrolysate proved to have a reproducible biostimulant effect after two years of treatment.

Wool keratin is an ideal source of proteins [28] compared to other protein bioresources and has been shown to biostimulate plant growth.

ATR-FTIR spectroscopy was used to compare the biochemical profile of grassland plant species related to land-use intensity [29] to determine how nitrogen fertilisation affects FTIR spectra in *Physalis* L. species [31], how a protein based biostimulant influences maize development [28], and even for rapid diagnosis of nitrogen status in rice from the amide I to amide II band intensity ratios [30]. Our study led to comparable results showing the potential of using ATR-FTIR data to monitor the influence of various keratin hydrolysates in *Zea mays* development. Improvement in plant growth due to biostimulant activity of ESPE and VALKE hydrolysates compared with the control was emphasised from increased concentrations in protein, carbohydrates, and phenolic derivatives directly related to increased spectral band intensity. Three different structures and compositions of keratin enzymatic hydrolysates, but also mesoscale properties like particle sizes showed biostimulant activity on *Zea mays*. ESPE hydrolysates showed the most important modifications in ATR spectra toward the control and VALKE hydrolysates in correlation with the experimentally proven performance on maize development, which can be attributed to the role of amino acids and oligopeptides as signal molecules in regulating the physiological mechanisms of growth and adaptation [31]. More thorough studies are necessary to infer the influence and mechanisms of enzymatic keratin hydrolysate biostimulant activity in *Zea mays* plant via hormone-like or antioxidant activity.

## 5. Conclusions

Enzymatic hydrolysis of wool alkaline keratin hydrolysate using three types of proteases showed the potential of keratin molecule tailoring for bioactive additive preparation. The evaluation of enzymatic keratin hydrolysate composition, molecular weight, functional groups, secondary molecule structure, and fungicide activity allowed us to understand the bioactive behaviour of different keratin-based products.

Biostimulant properties for maize plant foliar fertilisation were attributed to protein fractions with lower molecular weight with still organised molecules, rich in organic nitrogen, with high free amino acid content and antifungal protection capacity.

## Figures and Tables

**Table 1:** Physico-chemical characteristics of keratin hydrolysates.

Characterisation	SD ±	Samples			
		HK	PRO	ESPE	VALKE
Dry substance, %	0.35	9.68	10.60	9.26	9.24
Total ash, %	0.24	15.28	13.21	15.12	14.61
Total nitrogen, %	0.34	11.78	12.64	13.07	12.12
Protein substance, %	0.34	71.39	76.60	79.20	73.45
pH, pH units	0.10	7.49	7.37	7.50	6.99
Aminic nitrogen, % (% at protein substance)	0.05	0.86	1.01	0.91	1.21
Cysteine, %	0.03	0.95	3.38	2.18	1.91
Cystinic sulphur, %	0.03	0.25	0.90	0.58	0.51
Mw, mol/g	-	38,099	35,112	12,400	3758
Mn	-	38,005	29,030	12,397	3586
Polydispersity index		1	1.21	1	1.05
Free amino acids, % (% at protein substance)	-	-	-	4.9	4.3

**Table 2:** Amide I band deconvolution for the keratin samples.

Sample	β-Sheets (%)	Random Coils (%)	α-Helix (%)
HK	64.71	-	35.28
PRO	74.54	-	25.44
ESPE	45.30	28.15	26.53
VALKE	27.66	45.20	27.11

**Table 3:** The antifungal activity of keratin hydrolysates against *Fusarium oxysporum* ATCC 48112.

Sample	Results, (CFU/mL)	Reduction (%)	Log_10_ Reduction
Inoculums Concentration	T_0_ = 6.8 × 10^3^	-	-
HK	T_24_ = 0	100%	3.83
PRO	T_24_ = 0	100%	3.83
ESPE	T_24_ = 0	100%	3.83
VALKE	T_24_ = 1	99.99%	3.83

**Table 4:** Assignments of main vibrational bands (cm^−1^) of maize leaves.

Bands	ν_NH_	ν_CH_	ν_CH_	ν_C=O Ester_	Amide I	Amide II	δ _CH_	δ _CH_	Amide III	ν_C–O _ _Ester_	ν_C–O _ _Polysach_	ν_C–O _ _Polysach_	Fingerprint Region Bands		
Control	3341	2917	2849	1714	1640	1555	1462	1424	1259	1157	1048	1034	729	719	664
ESPE	3353	2917	2849	1715	1640	*	1463	1422	1243	1155	1048	1034	729	719	661
VALKE	3352	2917	2849	1715	1642	1553	1463	1416	1258	1156	1046	1034	729	719	661

* Amide II band is superposed with the amide I band and appears as a shoulder.

## Data Availability

This study did not report any data.
